# Individual ZnO–Ag Hybrid Nanorods for Synergistic Fluorescence Enhancement Towards Highly Sensitive and Miniaturized Biodetection

**DOI:** 10.3390/nano15080617

**Published:** 2025-04-17

**Authors:** Marion Ryan C. Sytu, Jong-in Hahm

**Affiliations:** Department of Chemistry, Georgetown University, 37th and O Sts. NW., Washington, DC 20057, USA

**Keywords:** semiconductor-metal, ZnO–Ag hybrid, hybrid nanorod, ZnO nanorod, Ag nanorod, biosensor, biodetection, fluorescence detection

## Abstract

Hybrid nanostructures can be engineered to exhibit superior functionality beyond the level attainable from each of the constituent nanomaterials by synergistically integrating their unique properties. In this work, we designed individual hybrid nanorods (NRs) of ZnO–Ag in different heterojunction configurations where each hybrid NR consists of a single ZnO NR forming a junction with a single Ag NR. We subsequently employed the ZnO–Ag hybrid NRs in the fluorescence detection of the model chemical and biological analytes, rhodamine 6G (R6G), and tumor necrosis factor-α (TNF-α), that undergo simple as well as more complex immunoreaction steps on the hybrid NRs. We determine how parameters such as the analyte concentration, ZnO–Ag heterojunction configuration, and NR length can influence the fluorescence signals, enhancement factors (EFs), as well as changes in EFs (%EFs) at different positions on the hybrid NRs. We provide much needed insights into the fluorescence enhancement capability of single hybrid NR systems using a signal source located external to the NRs. Moreover, we identify key consideration factors that are critical to the design and optimization of a hybrid NR platform for achieving high signal enhancements. We show that higher EFs are consistently observed from the junction relative to other positions in a given hybrid NR, from the end–end relative to other heterojunction configurations, and from longer than shorter ZnO NRs. Our research efforts demonstrate that the synergistic interplay of the two component NRs of ZnO and Ag escalates the fluorescence detection capability of the ZnO–Ag hybrid NR. A superior enhancement level surpassing those attainable by each component NR alone can be obtained from the hybrid NR. Hence, our work further substantiates the potential utility of individual semiconductor-metal hybrid NRs for highly miniaturized and ultra-trace level detection, especially by leveraging the critical consideration factors to achieve a higher detection capability.

## 1. Introduction

Hybrid nanostructures, comprised of distinct nanomaterials, can achieve performance levels beyond the capabilities of individual materials alone, offering improved functionality in many technological applications [1,2,3,4,5,6,7]. For example, hybrid nanostructures of semiconductor-metal have been demonstrated for their complementary properties that enhance light-matter interactions and permit control over optical functionalities [8,9,10,11,12,13]. Their synergistic benefits have been reported for the usage in biosensing [14,15], photocatalytic [9,16], and photonic [8,17] applications. An exemplar semiconductor nanomaterial well-known for its exceptional optical properties is ZnO nanorods (NRs), whose utility has been demonstrated in a wide range of applications from biosensing [18,19,20], to photocatalysis [21,22], and to energy conversion [23,24]. In addition to employing ZnO by itself, strategies to create hybrid nanostructures of ZnO for attaining even higher NR performance have been explored by integrating ZnO NRs with metal nanoparticles (NPs) or thin films of Au and Ag [25,26,27]. So far, these research endeavors have been centralized on ensembles of nanomaterials instead of single nanomaterials [25,26,27]. Moreover, most of these efforts have focused on the optical signals generated by the nanomaterials themselves, including the near-band-edge emission (NBE) of the ZnO NRs and the localized surface plasmon resonance (LSPR) of the Au or Ag NPs. These signals have been exploited in photoluminescence [28,29] and photodetection applications [17,30]. However, as frequently encountered in many biosensing applications, analyte detection can involve trace-level optical signals emitted by external sources instead of the inherent signals generated from the nanomaterial themselves [31,32,33,34,35,36]. At the same time, the ongoing pursuit for highly compact biosensors further necessitates the use of individual nanomaterials rather than ensembles of nanomaterials as active sensing elements [37,38]. Yet, our current understanding of individual hybrid systems of ZnO–metal remains very limited, particularly regarding optical signals generated by an independent source placed in the vicinity of a hybrid NR.

Nevertheless, individual ZnO NRs have been examined in a series of fluorescence measurements to determine their detection capability by evaluating optical signals produced by fluorophores or fluorophore-conjugated analytes [18,19,20,39,40,41,42]. A unique optical phenomenon termed fluorescence intensification on nanorod ends (FINE) has been revealed [42]. This phenomenon stems from the ability of individual ZnO NRs to effectively concentrate, guide, direct, and intensify light from a nearby source owing to the large refractive index, high shape anisotropy, and reduced dimensionality of the ZnO NR [18,43]. Such subwavelength waveguiding capabilities of individual ZnO NRs have proven valuable, especially in fluorescence-based biodetection, by being able to effectively confine the bioanalyte signals within the NR’s optical cavity, finally radiating out the well-guided and highly intensified signals through the NR ends [19,20,39,40,41]. Despite these endeavors, ZnO–metal hybrid systems have never been investigated for such potential in fluorescence detection. The degree of signal enhancement attainable using a hybrid system consisting of a ZnO NR and a metal NR, compared to that by either the ZnO NR or the metal NR alone, remains unknown. Hence, uncovering the synergistic interplay between a ZnO NR and a metal NR at the single ZnO–metal hybrid level will be crucial. The anticipated knowledge will be important for facilitating the miniaturization of the hybrid sensing technology that can enable a low volume, high throughput analysis [42,44,45]. It will also be essential for acquiring accurate signal response behaviors from individual nanomaterials, whose characteristics can otherwise be concealed in the averaged responses of ensemble-level measurements [42]. Additionally, it is critical to spatially resolve the measured fluorescence intensity as a function of the different positions within a given hybrid NR and to establish exact relationships between them [46]. This capability will benefit the improvement of the hybrid NR’s detection sensitivity by guiding the optimal design of the hybrid NR platform as well as the strategic analyte placement on the platform.

In this study, we created individual hybrid NR structures composed of ZnO–Ag for use in both proof-of-concept and bioapplication-ready fluorescence detection. Each hybrid NR is made up of a single ZnO NR connected to a single Ag NR, while the optical signals are emitted from an external source, either by standalone fluorophores or fluorophore-conjugated biomolecules, located in the vicinity of the hybrid NR. As a proof-of-concept, the optical signals from a model fluorophore of rhodamine 6G (R6G) are spatially resolved and characterized along individual ZnO–Ag hybrid NRs. We systematically elucidate signal enhancement factors (EFs) as a function of distinct positions within a given hybrid NR as well as their dependence on the fluorophore concentration, hybrid junction configuration, and NR length. We further demonstrate the application of individual ZnO–Ag hybrid NRs in biosensing by successfully carrying out the fluorescence detection of an immunocomplex formed in a protein biomarker reaction. Tumor necrosis factor-α (TNF-α) is chosen in our study due to its implicated role in inflammatory and autoimmune diseases such as kidney injury, psoriasis, and rheumatoid arthritis [19,47]. Our study reveals, at the individual hybrid NR level, the exact spatial distribution characteristics of the bioanalyte fluorescence signals enhanced by the hybrid NR to a level beyond what each component NR can offer alone. We identify critical aspects such as the junction position, end–end heterojunction configuration, and longer ZnO NR that need to be considered for the design of a hybrid NR platform to maximize the signal enhancement. Our efforts may advance the development of miniaturized ZnO–Ag and other similar hybrid biosensors by providing critical insights to best capitalize on the synergistic properties of the nanomaterials for enhanced fluorescence detection.

## 2. Materials and Methods

ZnO NRs were synthesized in a home-built, horizontal tube furnace via chemical vapor deposition (CVD). A 1:2 weight mixture of 99.999% ZnO and 99% graphite powders (Alfa Aesar Inc., Tewksbury, MA, USA) served as the source material to grow the ZnO NRs. A colloidal solution of 20 nm Au NPs (Ted Pella, Inc., Redding, CA, USA) was deposited onto a Si wafer (Silicon Quest International Inc., San Jose, CA, USA) as the growth catalyst. The source material was contained in a quartz boat at the center of the tube furnace, while the Si wafer with Au catalysts was positioned 15 cm downstream. The tube furnace was heated to 950 °C at a rate of 15 °C/min and maintained at this temperature up to an hour, under a constant Ar flow of 100 standard cubic centimeters per minute. Ag NRs were synthesized via a modified method with a polyvinylpyrrolidine (PVP) to Ag molar ratio of 5.5:1 [48]. Briefly, 100.1 mg PVP (M_w_ = 55,000; Sigma Aldrich, St. Louis, MO, USA) and 1.7 mg NaCl were dissolved in 2 mL ethylene glycol (EG) from Fisher Scientific Inc (Pittsburg, PA, USA). The solution was then heated to 170 °C while stirring at 1000 rpm for 30 min. Separately, a solution of AgNO_3_ in EG was prepared by dissolving 27.3 mg AgNO_3_ in 2 mL EG. This solution was subsequently added dropwise to the PVP solution at a rate of 5.5 mL/h under continuous stirring. The final mixture was maintained at 170 °C for an additional 30 min. The as-synthesized Ag NRs were drop-casted onto a clean Si wafer and incubated for 5 min, after which the excess solution was rinsed off with DI water. The as-grown ZnO NRs were sonicated in ethanol and subsequently deposited onto the Si wafer pre-coated with Ag NRs. The morphological characterization of the resulting ZnO–Ag NR hybrids was conducted with a FEI/Philips XL 20 (Hillsboro, OR, USA) scanning electron microscope (SEM) operated at 20 keV.

Rhodamine 6G (R6G) solutions of 25, 250 and 2500 ng/mL were prepared in deionized (DI) water via the serial dilution of R6G powder obtained from Sigma Aldrich (St. Louis, MO, USA). Recombinant tumor necrosis factor-alpha (TNF-α) from R&D Systems (Minneapolis, MN, USA) was used to prepare TNF-α standard solutions at concentrations of 0.1, 1, 10, 100 pg/mL through serial dilutions in DI water. Unlabeled monoclonal anti-human TNF-α antibody (clone 6401, R&D Systems, Minneapolis, MN, USA) was employed as a primary capture antibody in our sandwich-type immunoassay reactions. Lyophilized bovine serum albumin (BSA) was procured from VWR Scientific, Inc. (West Chester, PA, USA) and reconstituted according to the manufacturer’s instructions. Polyclonal anti-human TNF-α antibody (AB 210-NA, R&D Systems, Minneapolis, MN, USA) was conjugated with a fluorophore, Alexa488, using the Monoclonal Antibody Labeling kit (Invitrogen Molecular Probes, Eugene, OR, USA) following the manufacturer’s protocol. The hybrid NRs of ZnO–Ag on Si were incubated with a 20 μL aliquot of the R6G solution of a desired concentration for 15 min. Following incubation, the sample was rinsed with DI water and dried using N_2_ gas to yield R6G-coupled, ZnO–Ag hybrid NRs. In a separate set of experiments, a 15 μL aliquot of 1 μg/mL solution of the unlabeled TNF-α primary antibody was deposited for 15 min onto a hybrid NR platform of ZnO–Ag, followed by thorough rinsing with DI water. To block unoccupied binding sites, the hybrid NR platform was incubated with 15 μL of 1% (*w*/*v*) BSA solution for 20 min, after which any unbound BSA was rinsed off with DI water. Next, a 15 μL aliquot of the TNF-α standard solution of a desired concentration was added to the NR hybrids and incubated for 15 min before rinsing with DI water. Lastly, the hybrid NR platform was incubated with 15 μL of 1.5 μg/mL solution of the Alexa488-labeled TNF-α secondary antibody for 30 min. The sample was then washed with DI water and gently dried with a stream of N_2_ gas. All reactions were conducted at room temperature in a humidity-controlled chamber protected from light. The aforementioned immunoassay scheme results in a sandwich-type immunocomplex of Alexa488-TNF-α, consisting of unlabeled TNF-α capture antibodies/TNF-α/Alexa488-labeled TNF-α secondary antibodies, on the hybrid NRs of ZnO–Ag.

Fluorescence measurements were performed with a Zeiss Axio Imager A2M microscope (Carl Zeiss, Inc., Jena, Germany) equipped with an AxioCAM HRm digital camera (Carl Zeiss, Inc., Jena, Germany). A 120 W Hg lamp (X-Cite 120Q, Carl Zeiss, Inc., Jena, Germany) served as the source for fluorescence excitation. Emission from both the R6G and Alexa488-TNF-α immunocomplex on the ZnO–Ag hybrid NRs was captured with an EC Epiplan-NEOFLUAR objective lens (50×, numerical aperture of 0.8) using a spectroscopic filter cube (λ_excitation,R6G_ = 510–540 nm, λ_emission,R6G_ = 575–640 nm; λ_excitation,Alexa488_ = 450–490 nm, λ_emission,Alexa488_ = 510–540 nm) at a 5 s exposure. Fluorescence data acquisition and analysis were performed using Zeiss AxioVision Rel. 4.8, Image J 1.54, and Origin 9 software packages.

## 3. Results and Discussion

**Figure 1:**
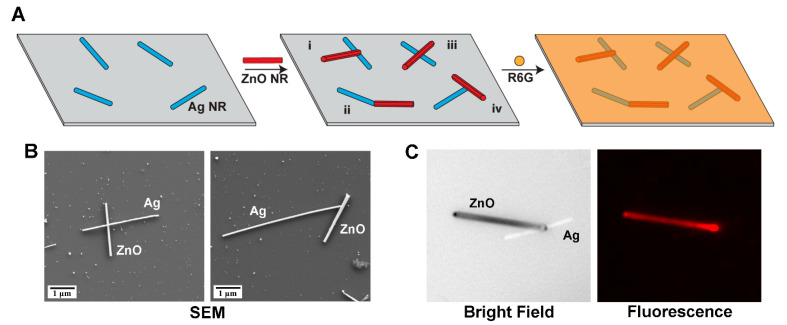
(**A**) The scheme depicts the fabrication process of R6G-coupled ZnO–Ag hybrid NRs. The hybrids were created by dispersing ZnO NRs onto a Si wafer pre-treated with Ag NRs. The four different heterojunction configurations shown as i, ii, iii, and iv were commonly observed from the resulting hybrid NRs of ZnO–Ag. A desired concentration of R6G solution was subsequently introduced to the hybrid NR platform. (**B**) Representative SEM images of pristine ZnO–Ag hybrid NRs are displayed. (**C**) Representative optical images obtained from R6G-modified hybrid NRs of ZnO–Ag are shown. The bright field (**left**) and the corresponding fluorescence (**right**) panels were obtained from the same ZnO–Ag hybrid NR when the sample was treated with 25 ng/mL of R6G solution. Under this low analyte concentration, discernable fluorescence signals were detected predominantly in the ZnO NR region. The emission from R6G is pseudo-colored red in the fluorescence image.

Figure 1A illustrates the fabrication process of R6G-modified, hybrid NRs of ZnO–Ag on a Si wafer. The hybrid samples were created by dispersing ZnO NRs onto a Si wafer that was pre-treated with Ag NRs. Four different heterojunction configurations are commonly observed from the resulting NR hybrids of ZnO–Ag, as represented by i, ii, iii, and iv in Figure 1A. These will be discussed in more detail later in the paper. A desired concentration of R6G solution was subsequently introduced to the hybrid NR platform of ZnO–Ag. Figure 1B presents representative SEM images of pristine hybrid NRs of ZnO–Ag. Each hybrid consists of a single ZnO NR connected to a single Ag NR by forming a ZnO–Ag heterojunction in one of the four ways. Figure 1C displays representative optical images obtained from the hybrid NRs of ZnO–Ag after the R6G deposition. The bright field (left) and fluorescence (right) images in Figure 1C were taken from the same hybrid sample after treating it with 25 ng/mL of R6G solution. At this low analyte concentration, discernable fluorescence signals were detected mainly from the ZnO NR portion of the ZnO–Ag hybrid NRs. Under the same experimental setting, the analyte signals were unnoticeable on the Ag NR segment. This can be evidenced in the pseudo-colored fluorescence data in Figure 1C.

ZnO NRs as stand-alone materials have previously been shown to enhance optical signals through subwavelength waveguiding [18,20,39,40,42]. Our data confirmed that the emitted signals from R6G can be effectively coupled to and guided along the ZnO NR when it is constructed into a ZnO–Ag hybrid NR structure as well. As for the Ag NR segment, plasmonic metals such as Ag are known for their ability to give rise to metal-enhanced fluorescence (MEF) [27,49,50]. Despite this, this mechanism did not appear to take effect for the low concentration of R6G directly placed on the Ag NR surface. It is worthwhile to note that the detected fluorescence intensity was exceptionally strong at the junction, where the NR segments of ZnO and Ag cross each other. In fact, the junction position marked the site with the most intense R6G signal among all positions of the hybrid NR. Given the considerably higher fluorescence intensity observed on the ZnO NR segment relative to the Ag NR portion, the subwavelength waveguiding capabilities of the ZnO NR may play a critical part in producing the enhanced signal at the junction. This contribution may be synergistically escalated at the hybrid junction due to the presence of Ag that can act as a plasmonically active substance to further concentrate and intensify the guided R6G signals. Additionally, charge transfer processes occurring at a semiconductor-metal interface are known to reduce electron-hole recombination [1,51]. Similar processes may take place at the ZnO–Ag hybrid junction and channel more energy into radiative than nonradiative pathways. This, in turn, may mitigate fluorophore quenching, promote efficient fluorophore excitation, and contribute to greater fluorescence emission.

**Figure 2:**
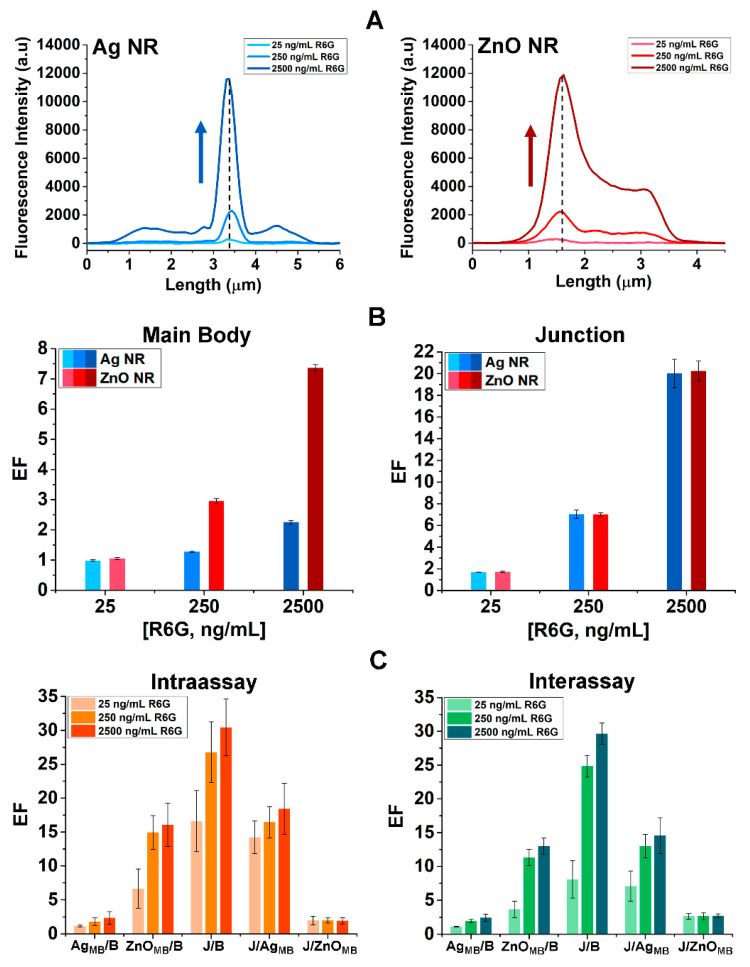
Fluorescence intensity profiles of R6G were analyzed along the various positions on individual ZnO–Ag hybrids. (**A**) Representative plots of fluorescence intensity measured along the Ag NR (blue, left) and the ZnO NR (red, right) of a ZnO–Ag hybrid NR are presented. The dashed lines mark the position of the junction where the ZnO and Ag segments intersect in a hybrid NR. The upward arrows indicate increasing R6G concentration. (**B**) The bar graphs display the average enhancement factor (EF) values obtained along the main body (left plot) and at the junction (right plot) under the R6G concentrations of 25, 250, and 2500 ng/mL. The blue and red bars correspond to the EF values determined from the hybrid NR portion of Ag and ZnO, respectively. (**C**) The bar graphs show the average EF values corresponding to the ratios of Ag_MB_/B, ZnO_MB_/B, J/B, J/Ag_MB_, and J/ZnO_MB_ obtained from the intraassay (orange, left) and interassay (green, right) measurements.

We systematically examined the measured R6G fluorescence intensity as a function of R6G concentration as well as position within a ZnO–Ag hybrid NR. Figure 2A shows representative plots of fluorescence intensity spatially resolved along the Ag NR segment (left panel) as well as that of the ZnO NR segment (right panel) in ZnO–Ag hybrid NRs. Dashed lines in both panels indicate the junction position where the two component NRs of ZnO and Ag intersect. In both plots of Figure 2A, the intensities measured at all positions along each NR generally increased with R6G concentration. Notably, the intensity at the hybrid junction was significantly higher than the signals observed along the ZnO NR main body (ZnO_MB_) and even more pronounced compared to those along the Ag NR main body (Ag_MB_). Furthermore, it is clear from Figure 2A that the junction exhibited a greater degree of fluorescence intensity change. This intensity change became increasingly more pronounced with higher R6G concentrations to the point that it surpassed the combined intensities of the ZnO_MB_ and Ag_MB_. The bar graphs in Figure 2B correspond to the enhancement factors (EFs). EF values were calculated by taking the ratio of the analyte fluorescence intensity measured either at the main body or the junction to that of the background area (B) on the Si substrate. The EFs calculated from the main body intensities of the component NRs are shown in the left panel of Figure 2B. The blue and red bars belong to the EF values of the Ag_MB_ and ZnO_MB_, respectively. The EF values calculated from the junction intensity are displayed in the right panel of Figure 2B. The bar graphs of ZnO_MB_, Ag_MB_, and junction show that the EF values increased with R6G concentration at all positions of the hybrid NR. It was also found that the EFs on the ZnO_MB_ are consistently higher compared to those on the Ag_MB_. At the same R6G concentration of 2500 ng/mL, the EF on the ZnO_MB_ was determined to be ~7 whereas the EF on the Ag_MB_ was only ~2. This trend became more noticeable with larger R6G concentrations. The EFs at the junction position were revealed to be significantly higher than those on the ZnO_MB_, with even greater enhancement relative to the Ag_MB_. At the R6G concentration of 2500 ng/mL, the EF at the junction of the ZnO–Ag hybrid NR reached ~20. These results quantitatively show that the fluorescence intensity observed at the junction is not just the simple sum of the fluorescence intensities from the two component materials that constitute the ZnO–Ag hybrid NR.

The different degrees of fluorescence enhancement from the R6G-modified ZnO–Ag hybrids were further substantiated by conducting intra- and inter-assays and spatially resolving the fluorescence signals at different positions within the same set of hybrid NRs as well as across multiple hybrid NRs. Figure 2C displays the resulting bar graphs of the average EF values determined from various positions on the ZnO–Ag hybrid NRs. The relative R6G intensities between two positions of comparison were calculated and included in the bar graphs to show the EFs of Ag_MB_ relative to B (Ag_MB_/B), ZnO_MB_ to B (ZnO_MB_/B), junction to B (J/B), junction to Ag_MB_ (J/Ag_MB_), and junction to ZnO_MB_ (J/ZnO_MB_). The left panel of Figure 2C corresponds to the results from the same set of ZnO–Ag hybrid NRs exposed to increasing R6G concentrations. The right panel of Figure 2C belongs to the data obtained from different hybrid samples under varying R6G concentrations. These measurements are referred to as intraassay for the former and interassay for the latter. Both the intra- and inter-assay results exhibited the same trend, with the EF values for Ag_MB_/B, ZnO_MB_/B, J/B, and J/Ag_MB_ consistently increasing with R6G concentration. Across all R6G concentrations tested, the junction signals were considerably higher than the signals from any other position on the hybrid NRs. The J/B ratios in the bar graphs were consistently higher than the values of ZnO_MB_/B and even greater than those of Ag_MB_/B. At the analyte concentration of 2500 ng/mL, the observed enhancement relative to the background reached 3-fold on the Ag main body, 13-fold on the ZnO main body, and 30-fold at the junction of the hybrid NR. As the fluorescence intensity at the junction tended to increase more significantly than that on the Ag main body, the corresponding EFs of J/Ag_MB_ increased with higher R6G concentration, although the change was not as pronounced as the J/B case. In contrast, the EFs for J/ZnO_MB_ showed minimal variations with R6G concentration. This was because, on both positions of the junction and the ZnO main body, the measured fluorescence signals were proportional to the analyte concentration. In addition, the J/ZnO_MB_ ratios were consistently greater than one from all the data presented in Figure 2C. This indicated that the correlation found between the measured fluorescence intensity and R6G concentration is much stronger at the junction relative to the ZnO main body position. The intra-and inter-assays were extended to ~180 hybrid NRs of ZnO–Ag and the main trends discussed above were repeatedly observed. When the junction signals were compared to the ZnO and Ag main body signals, the EFs of J/ZnO_MB_ and J/Ag_MB_ in our intra- and inter-assays were found to be approximately 4-fold and 15-fold, respectively. Hence, the formation of a hybrid structure benefited the fluorescence detection capability of the Ag NR substantially more than the ZnO NR. The fluorescence enhancement attained at the junction of a ZnO–Ag hybrid NR exceeds the simply additive detection level from the two component nanomaterials, allowing synergistically enhanced fluorescence detection through the formation of the hybrid NR.

**Figure 3:**
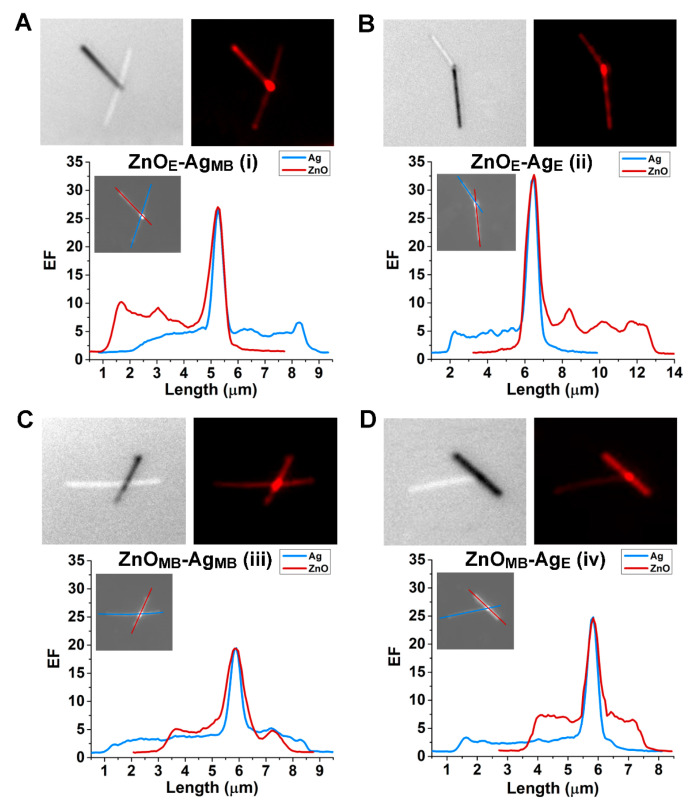
Fluorescence intensity profiles of R6G were examined on the four commonly found heterojunction configurations of the ZnO–Ag hybrid NRs. The analyte fluorescence intensity was spatially resolved along the various positions in individual ZnO–Ag hybrids and presented for the heterojunction case of (**A**) ZnO_E_-Ag_MB_, (**B**) ZnO_E_-Ag_E_, (**C**) ZnO_MB_-Ag_MB_, and (**D**) ZnO_MB_-Ag_E_. In (**A**–**D**), the top panels display bright field images on the left and pseudo-colored fluorescence images on the right for each ZnO–Ag hybrid NR. The bottom panels show the EF plots as a function of the position along the two component NRs in the hybrid. The blue and red traces were taken along the NR portion of Ag and ZnO, respectively. The inset in each plot depicts the line analysis section for the NR segment of Ag and ZnO to obtain the blue and red traces in the EF plots.

We subsequently investigated the effects of the ZnO–Ag heterojunction configurations on fluorescence enhancement by examining R6G-modified ZnO–Ag hybrid NRs at the individual hybrid level. Figure 3 presents a representative set of data obtained from the four different cases of ZnO–Ag configuration in a hybrid, as previously illustrated as i through iv in Figure 1A. The data shown in Figure 3A–D belong to the heterojunction configurations of one end of a ZnO NR connected to the main body of a Ag NR (ZnO_E_-Ag_MB_), one end of a ZnO NR connected to one end of a Ag NR (ZnO_E_-Ag_E_), the main body of a ZnO NR connected to the main body of a Ag NR (ZnO_MB_-Ag_MB_), and the main body of a ZnO NR connected to one end of a Ag NR (ZnO_MB_-Ag_E_), respectively. In Figure 3A–D, the top panels display bright field images on the left and the corresponding fluorescence images on the right. The bottom panels show EF values plotted as a function of the position along each component NR within a hybrid, with the blue and red traces corresponding to the Ag and ZnO segments of a given hybrid NR, respectively. The section of the line analysis used to generate the blue and red traces is also illustrated in the inset in each plot. It is clear, from all fluorescence data in Figure 3, that enhanced fluorescence intensities were detected at the junction compared to those observed on the ZnO and Ag main bodies. It is also evident across all plots that the maximum EF was found at the junction location. Among the different configurations, the junction EF value was the highest for the case of ZnO_E_-Ag_E_ in Figure 3B and the lowest in the case of ZnO_MB_-Ag_MB_ in Figure 3C. Hence, the analyte signal enhancement at the junction was maximized in the hybrid configuration of ZnO_E_-Ag_E_, in which case the formation of the heterojunction involved the end facets of the two component NRs. In contrast, when only the main body facets were involved in the heterojunction formation, the enhancement at the junction was found to be at a minimum, as in the case of ZnO_MB_-Ag_MB_. The other two configurations of ZnO_E_-Ag_MB_ in Figure 3A and ZnO_MB_-Ag_E_ in Figure 3D correspond to the heterojunction cases formed by an end facet of one material connected to a main body facet of the other material. In these cases, intermediate levels of enhancement were observed at the junction, with the EF values comparable between the two cases. Our data suggest that the ZnO–Ag hybrid NRs with their junctions formed in an end–end fashion contribute to the highest junction EFs attainable from the different heterojunction configurations.

**Figure 4:**
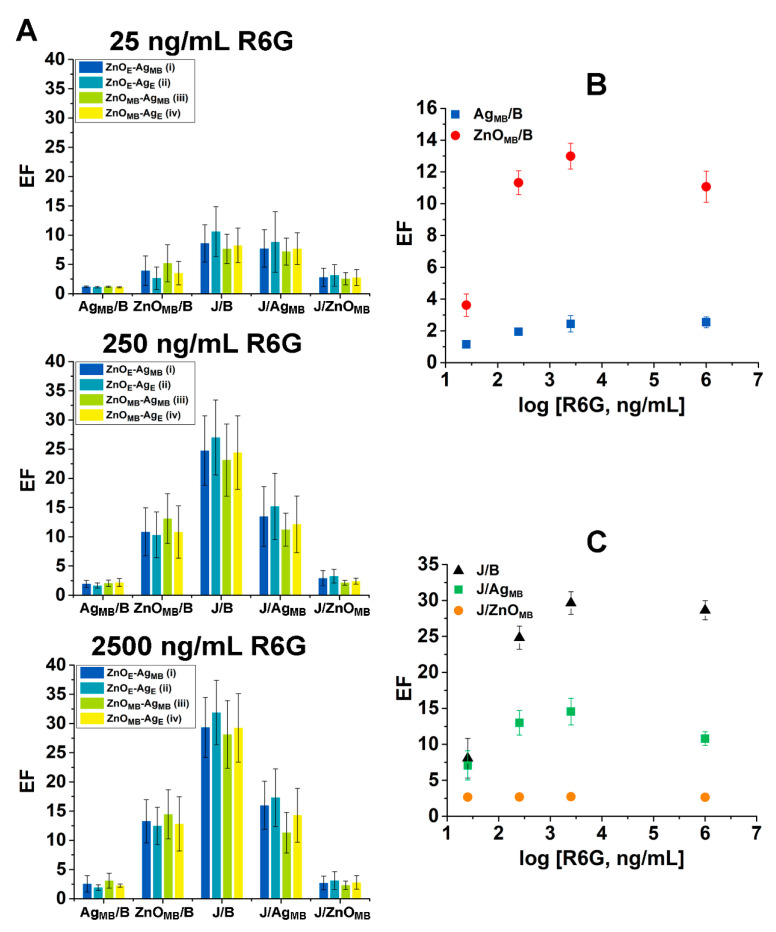
EFs were determined for different ZnO–Ag hybrid configurations as well as for various R6G concentrations. (**A**) The bar graphs present the average EF values corresponding to Ag_MB_/B, ZnO_MB_/B, J/B, J/Ag_MB_ and J/ZnO_MB_ for the heterojunction configuration cases of ZnO_E_-Ag_MB_ (blue), ZnO_E_-Ag_E_ (teal), ZnO_MB_-Ag_MB_ (yellow green) and ZnO_MB_-Ag_E_ (yellow). The top, middle, and bottom panels correspond to the data when 25, 250, and 2500 ng/mL of R6G was used, respectively. (**B**,**C**) The plots show the average EF values as a function of R6G concentration. Data in (**B**) belong to Ag_MB_/B (blue square) and ZnO_MB_/B (red circle), whereas those in (**C**) show J/B (black triangle), J/Ag_MB_ (green square), and J/ZnO_MB_ (orange circle).

Figure 4A compares the different EFs among the four ZnO–Ag heterojunction configurations when the R6G concentration was varied from 25 (left), to 250 (middle), and to 2500 (right) ng/mL. The average EF values of J/B, J/Ag_MB_, and J/ZnO_MB_ were highest for the heterojunction configuration of ZnO_E_-Ag_E_ (data in teal), with the maximum junction enhancement attained under this end–end configuration. As for the main body signals, the ZnO_E_-Ag_E_ configuration yielded the lowest values of Ag_MB_/B and ZnO_MB_/B. These observations indicate that the end–end hybrid configuration results in analyte signals being highly concentrated at the junction, leaving much weaker signals on any of the main body positions. In contrast, the highest Ag_MB_/B and ZnO_MB_/B ratios along with lowest J/B, J/Ag_MB_ and J/ZnO_MB_ values were found from the heterojunction configuration of ZnO_MB_-Ag_MB_ (yellow green). For this hybrid configuration of main body–main body, the analyte signals seemed to be distributed over the main bodies of the two component NRs rather than being centralized at the junction. When it comes to the configuration case of end–main body such as those of ZnO_E_-Ag_MB_ (blue) and ZnO_MB_-Ag_E_ (yellow), all EFs of Ag_MB_/B, ZnO_MB_/B, J/B, J/Ag_MB_, and J/ZnO_MB_ produced intermediate values compared to the end–end or the main body–main body cases. Moreover, the aforementioned trends were consistently observed across all R6G concentrations in Figure 4A. These findings demonstrate the important role that the hybrid configuration may play in achieving the highest possible EFs.

The different degrees of fluorescence enhancement of the R6G-modified ZnO–Ag hybrid NRs were further scrutinized under various analyte concentrations. Figure 4B,C present the resulting data in the plots of average EFs as a function of R6G concentration when the concentration of R6G was varied in the range between 25 ng/mL and 1 mg/mL. Figure 4B displays the average EF values of Ag_MB_/B (data in blue square) and ZnO_MB_/B (red circle), while Figure 4C presents those for J/B (black triangle), J/Ag_MB_ (green square), and J/ZnO_MB_ (orange circle). The EFs generally rose with R6G concentration up to 2500 ng/mL. This is consistent with the earlier observations of higher fluorescence intensities detected on the main bodies as well as at the junction with increasing analyte concentration. The EF values under the very high concentration of 1 mg/mL seemed to either plateau or slightly decrease. This may stem from the well-known self-quenching behaviors of the fluorophores at high concentrations [52,53]. Unlike other data in Figure 4B,C, the J/ZnO_MB_ ratios remained largely constant with the analyte concentration. As discussed previously, the analyte concentration did not affect the J/ZnO_MB_ values which are the ratios of the two highly concentration-dependent intensities.

**Figure 5:**
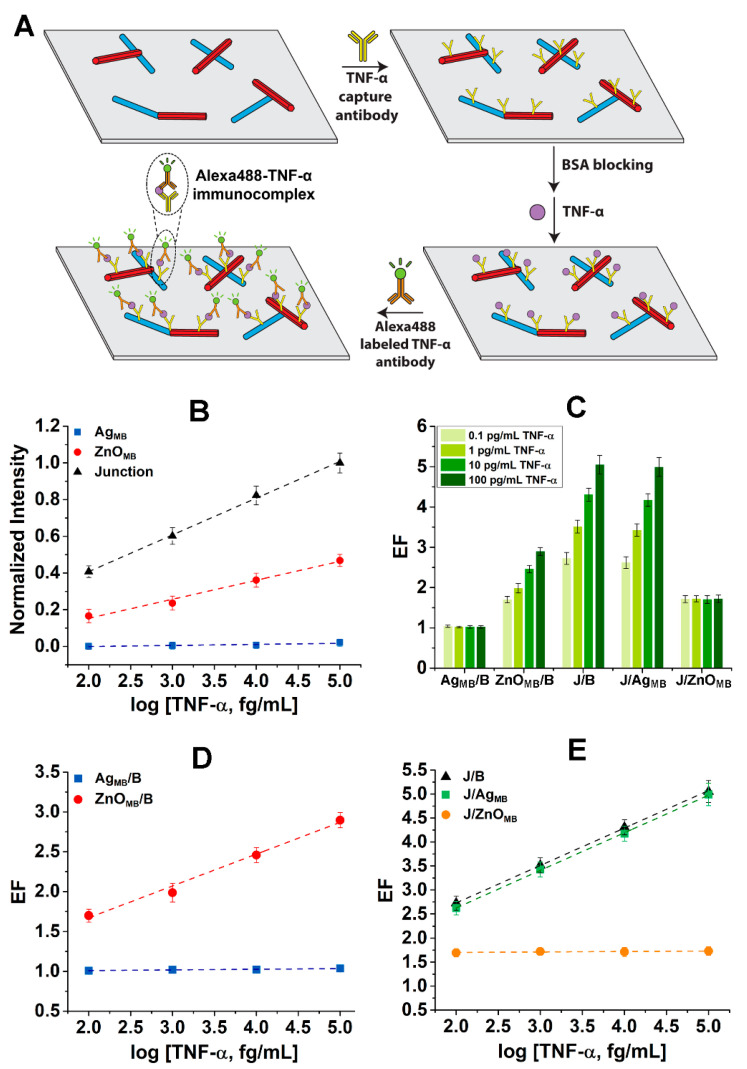
(**A**) The scheme illustrates the multistep TNF-α immunoassay reactions carried out on individual ZnO–Ag hybrid NRs. The immunoassay reactions led to the formation of Alexa488-TNF-α immunocomplex. (**B**) The plot displays normalized intensity with respect to TNF-α concentration for the junction (black triangle), Ag_MB_ (blue square), and ZnO_MB_ (red circle). (**C**) The bar graph shows the average EF values for Ag_MB_/B, ZnO_MB_/B, J/B, J/Ag_MB_, and J/ZnO_MB_ at varying TNF-α concentrations. (**D**,**E**) The plots show the average EF values as a function of TNF-α concentration. The data in (**D**) correspond to Ag_MB_/B (blue square) and ZnO_MB_/B (red circle), whereas those in (**E**) belong to J/B (black triangle), J/Ag_MB_ (green square), and J/ZnO_MB_ (orange circle). In (**B**,**D**,**E**), the dashed lines are the linear fits obtained through the data points.

Next, we assessed the possibility of employing ZnO–Ag hybrid NR platforms in more practical biodetection by using TNF-α as a model bioanalyte. An immunoassay protocol devised for the TNF-α detection on individual ZnO–Ag hybrid NRs is shown in Figure 5A. First, unlabeled TNF-α capture antibodies were adsorbed to ZnO–Ag hybrid NRs. The step of BSA blocking was followed afterwards to passivate any unbound surface sites on the hybrid NRs as well as the underlying Si substrate. TNF-α solution of a desired concentration was added in the subsequent step of the immunoassay. Finally, Alexa488-labeled, TNF-α secondary antibodies were introduced. These immunoreaction steps generated Alexa488-TNF-α immunocomplexes which consisted of unlabeled TNF-α capture antibodies/TNF-α/Alexa488-labeled TNF-α secondary antibodies on the hybrid NRs of ZnO–Ag.

The fluorescence intensities of the Alexa488-TNF-α immunocomplex on the ZnO–Ag hybrid NRs were then assessed when different TNF-α concentrations of 0.1, 1, 10, and 100 pg/mL were used in the sandwich-type immunoassays. The plot in Figure 5B presents normalized fluorescence intensity as a function of TNF-α concentration on the main body positions of Ag_MB_ (blue square), ZnO_MB_ (red circle), and at the junction position (black triangle). The fluorescence intensity linearly increased with the TNF-α concentration, both on the ZnO main body and junction positions. In addition, the slope of the regression line through the junction intensity data points was found to be steeper than that of the ZnO_MB_. This is an indication of a much stronger correlation between the fluorescence intensity at the junction and the TNF-α concentration relative to the concentration dependence of the fluorescence intensity observed on the ZnO main body. In contrast, the measured fluorescence intensities on the Ag main body showed a negligible correlation, keeping them close to the background values. Similar to what was discussed earlier for R6G, the fluorescence intensities measured on the ZnO NR segment of a given ZnO–Ag hybrid NR are much higher than those from the Ag segment for all TNF-α concentrations tested. Unlike the subwavelength waveguiding mechanism of the ZnO NR, the MEF mechanism for the Ag NR requires an optimal distance of approximately 5–20 nm between the fluorophore and the metallic nanostructures for any signal enhancement to take place [27]. After the multistep TNF-α reactions we used, the distance between the Alexa488 fluorophores and the Ag surface may not fall within the optimal range required to generate sufficient enhancement from the Ag segment. With the relatively strict distance requirements, similar enhancement trends for the Ag relative to the ZnO segment of the hybrid NR platforms are expected in other immunodetection settings as well.

The bar graph in Figure 5C is displayed to compare the degrees of fluorescence enhancement across various TNF-α concentrations. Overall, the key trends already discussed for the spatially resolved fluorescence signals on the ZnO–Ag hybrid NRs remained the same as those in the proof-of-concept experiments involving R6G. The fluorescence signals at the junctions of the ZnO–Ag hybrid NRs exhibited a linear relationship with TNF-α concentration, as did the dependence of the fluorescence intensities measured on the ZnO main body on the TNF-α concentration. The ZnO_MB_/B values grew with increasing TNF-α concentration. The J/ZnO_MB_ ratios remained unchanged for all TNF-α concentrations. Contrastingly, the Ag main body positions had negligible dependence on TNF-α concentration and the Ag_MB_/B values remained close to one, showing minimal changes despite the varying TNF-α concentrations. The values of J/B and J/Ag_MB_ were similar and rose with TNF-α concentration.

The abovementioned trends were further corroborated in the EF plots in Figure 5D,E. Figure 5D shows the average EF values for Ag_MB_/B (blue square) and ZnO_MB_/B (red circle) as a function of TNF-α concentration, while Figure 5E displays those for J/B (black triangle), J/Ag_MB_ (green square), and J/ZnO_MB_ (orange circle). The Ag_MB_/B ratios lay close to one, whereas the ZnO_MB_/B ratios increased linearly with TNF-α concentration. The J/B and J/Ag_MB_ ratios, whose EF values were similar to each other, showed a linear relationship with the TNF-α concentration as well. In contrast, the different TNF-α concentrations did not alter the J/ZnO_MB_ values. Furthermore, the much higher value of J/Ag_MB_ relative to J/ZnO_MB_ suggested that the ZnO–Ag hybrid formation provided a greater benefit to the Ag component than to the ZnO component.

For both R6G and TNF-α, the fluorescence intensities on the ZnO NR segment were found to be much larger than those on the Ag NR segment of the hybrid NRs. This makes it highly likely that a large portion of the signals observed at the hybrid junction is contributed by the analyte signals carried by the ZnO NR via subwavelength waveguiding. We have previously shown that longer ZnO NRs, rather than shorter ones, are more effective in fluorescence detection [20,39]. Hence, it may be plausible that the degree of signal enhancement in the ZnO–Ag hybrid NRs could also be influenced by the length of the ZnO NR portion. Therefore, we investigated whether the ZnO length in the hybrid NRs plays any role in the analyte signal enhancement observed at the junction relative to the main bodies. To systematically examine this, we determined %EF as a quantitative measure to evaluate the change in the enhancement level of the junction with respect to the main body positions. The %EF values were calculated using Equation (1), where EF_Junction_ represents the EF for the junction relative to the background, and EF_MB_ represents the EF for the main body of either component NR relative to the background.

(1) $$\%\mathrm{EF}=\frac{{\mathrm{EF}}_{\mathrm{Junction}\;}-\;{\mathrm{EF}}_{\mathrm{MB}}}{{\mathrm{EF}}_{\mathrm{MB}}}\;\times \;100\%$$

**Figure 6:**
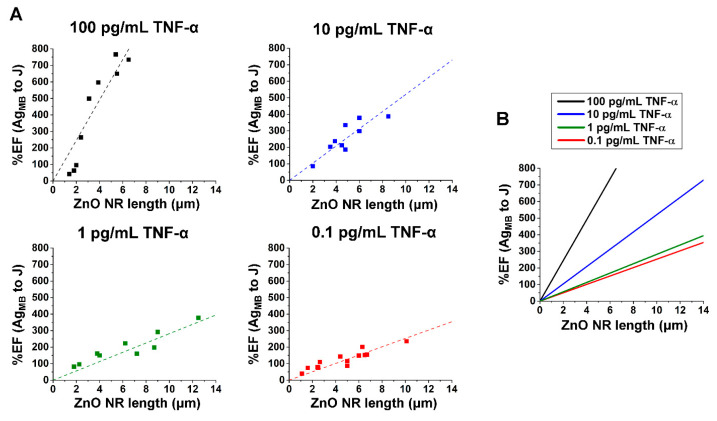
The effect of the ZnO NR length was correlated to the %EF changes determined from the junction and Ag main body of individual ZnO–Ag hybrid NRs. (**A**) The plots display %EF values determined from the junction and Ag main body versus ZnO NR length for the TNF-α concentrations of 100 (black), 10 (blue), 1 (green) and 0.1 (red) pg/mL. The dashed lines represent linear fits through the data points. (**B**) The plot shows the combined regressions from %EF change versus ZnO NR length for all TNF-α concentrations in (**A**). The slope of the regressions increases with higher TNF-α concentration.

The %EF changes calculated by comparing the junction to the Ag main body positions were then plotted against the ZnO NR length for varying TNF-α concentrations, as shown in Figure 6A. For all TNF-α concentrations, a linear relationship was found between the ZnO NR length and %EF change. Figure 6B is a plot of overlaid linear fits for all TNF-α concentrations shown in Figure 6A. The slopes of the combined regression lines indicate that the length dependence of %EFs is more pronounced at higher TNF-α concentrations. For example, at the ZnO NR length of 4 μm, the %EF change from the Ag main body to the junction was approximately 500% for the TNF-α concentration of 100 pg/mL, 200% for 10 pg/mL, and 100% for both 1 and 0.1 pg/mL. The differences in %EF change for each TNF-α concentration became even greater with longer ZnO NRs.

**Figure 7:**
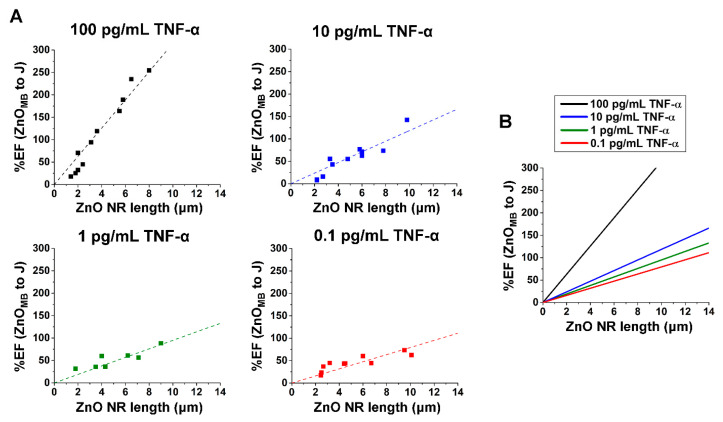
The effect of the ZnO NR length was correlated to the %EF changes determined from the junction and ZnO NR main body of individual ZnO–Ag hybrid NRs. (**A**) The plots display %EF changes of the junction to ZnO NR main body versus ZnO NR length for the TNF-α concentrations of 100 (black), 10 (blue), 1 (green) and 0.1 (red) pg/mL. The dashed lines represent the linear fits through the data points. (**B**) The plot shows the combined regressions from %EF change versus ZnO NR length for all TNF-α concentrations in (**A**). The slope of the regressions increases with higher TNF-α concentration.

The %EF changes between the junction and ZnO main body positions were also evaluated as a function of ZnO NR length across different TNF-α concentrations, as shown in Figure 7A. Similar to the %EF results obtained from comparing the junction to the Ag main body, the %EF changes from the ZnO main body to the junction were also proportional to ZnO NR length, as indicated by the regressions (dashed lines) in Figure 7A. Figure 7B displays the overlaid linear fits for all TNF-α concentrations. The correlation between %EF change and ZnO NR length was particularly pronounced at higher TNF-α concentrations, as reflected by the slopes of the regression lines. For instance, at the ZnO NR length of 4 μm, %EF values were determined to be approximately 125, 50, 35, and 25 for the TNF-α concentrations of 100, 10, 1, and 0.1 pg/mL, respectively. Across all TNF-α concentrations, the difference in %EF for ZnO_MB_ to J was more apparent for longer NRs. Additionally, the relatively higher %EF values of Ag_MB_ to J compared to those of ZnO_MB_ to J suggest that the formation of ZnO–Ag hybrid NRs can significantly enhance the otherwise weak signals observed on the Ag NR main body. As for the ZnO NR main body, the already substantial signals detectable from the ZnO segment seemed to make the relative gain from the hybrid formation less pronounced.

Our results suggest that the optical waveguiding nature of the ZnO NRs is the primary contributor to the analytes’ fluorescence signals observed at the ZnO–Ag hybrid NR junction. The considerably stronger fluorescence signals on the ZnO NR segment compared to the Ag NR segment indicate that a substantial portion of the junction signal originates from the ZnO component, owing to its excellent waveguiding properties. As exemplified in Figure 6 and Figure 7, the signal intensity at the junction increases with the length of the ZnO NRs. This is consistent with more efficient waveguiding in longer NRs. The ZnO NRs likely direct fluorescence emission toward the junction, where plasmonic effects and charge transfer interactions further enhance the signal by promoting fluorophore excitation, boosting radiative emission efficiency, and mitigating fluorescence quenching. Although the small surface area of the junction may limit the number of biomolecules that can directly bind at the site, much of the observed fluorescence at the junction originates from the signals of all biomolecules on the ZnO NRs that are guided along the NRs, rather than exclusively from those biomolecules located at the junction. Additionally, the confined geometry of the junction promotes high spatial localization of these signals, thereby improving detection sensitivity by concentrating both the effects of waveguided emission and plasmonic enhancement into a small, defined region.

## 4. Conclusions

In summary, we performed spatially resolved, fluorescence detection of R6G and TNF-α on ZnO–Ag hybrid NRs, at the individual hybrid NR level. We demonstrated that individual ZnO–Ag hybrid NRs can enhance the detection capability of not only chemical analytes following a simple reaction, but also bioanalytes after more complex reactions. We specifically assessed how factors such as analyte concentration, NR length, and heterojunction configuration can influence the fluorescence intensities detected at various key positions within a ZnO–Ag hybrid NR. We also revealed the exact relationship between the aforementioned factors and the EFs. The main finding from our study is that the employment of the ZnO–Ag hybrid NR in the fluorescence detection enabled the synergistic interplay between the ZnO NR’s subwavelength waveguiding and the Ag NR’s plasmonic effects. This permitted the detection of significantly increased analyte signals at the junction than what can be measured from each component material alone. The signal enhancement achieved by the ZnO–Ag hybrid NR, in terms of either EFs or %EFs, was dependent on the different positions along the hybrid NR. In particular, the fluorescence signals observed at the junction of the hybrid NR were notably higher than those at any other position in the hybrid NR. The measured signal intensities for a given hybrid NR, ranked from lowest to highest, were in the order of the Ag segment, the ZnO segment, and the junction. The junction intensity increased with higher analyte concentration. Moreover, the signal enhancement of the hybrid NR was correlated with the ZnO NR length, yielding greater %EFs in longer ZnO NRs. Additionally, we ascertained that the heterojunction configuration for the ZnO–Ag hybrid NR plays a role in signal enhancement. The intensity of fluorescence signals at the junction, ranked from highest to lowest, was observed from the two component NRs in the hybrid connecting in an end–end, end–main body, and main body–main body fashion. The ZnO–Ag hybrid NRs show great potential for use in biomedical diagnostics and point-of-care testing, particularly in applications requiring the detection of low-abundance biomolecules. Owing to the synergistically amplified fluorescence signals, our approach offered a detection sensitivity down to the pg/mL range. Additionally, the nanoscale architecture of the ZnO–Ag hybrid NRs enables a small sensor footprint, low-cost surface functionalization, and high multiplexing capabilities. This makes them well-suited for compact and cost-effective biosensing platforms. Although further studies are warranted to evaluate their performance characteristics in complex biological environments, our work successfully demonstrates the potentially useful functionality of the ZnO–Ag hybrid NRs in next-generation biosensing applications. The insights gained from our study may contribute to the strategic design and optimization of semiconductor-metal hybrid NR platforms, harnessing the synergistic optical properties of each component NRs for highly miniaturized and sensitive fluorescence detection.

## Data Availability

Data are contained within the article.
